# Herpes Zoster Ophthalmicus in a Healthy Toddler Fully Immunized Against Varicella-Zoster Virus: A Case Report and Review of Treatment Strategies in Children

**DOI:** 10.7759/cureus.33352

**Published:** 2023-01-04

**Authors:** Paraskevi Keramida, Marita Antoniadi, Eugenia Archimandritou, Stavroula Kostaridou, Patra Koletsi

**Affiliations:** 1 Pediatrics, Penteli Children's Hospital, Athens, GRC; 2 Ophthalmology, Penteli Children's Hospital, Athens, GRC

**Keywords:** treatment choices, hzo, paediatric uveitis, herpes zoster ophthalmicus, varicella-zoster virus, herpes zoster virus

## Abstract

Herpes zoster ophthalmicus (HZO) is a rare presentation of herpes zoster in children; however, it may become chronic and debilitating. The pathophysiology of HZO complications is not completely understood and may include virus virulence, vascular and neural inflammation and immune reactivity. Therefore, clinical experts suggest an antiviral agent combined with topical steroids, but treatment duration and the need for secondary prophylaxis, given the likelihood of recurrence, are not clearly defined. We present a complex case of HZO in a varicella zoster virus (VZV)-vaccinated toddler successfully treated with acyclovir and topical steroids. We also present a review of the relevant literature regarding the therapeutic management and long-term sequelae of HZO in children.

## Introduction

Herpes zoster ophthalmicus (HZO) is a rare complication of herpes zoster (HZ) and may follow primary varicella-zoster virus (VZV) infection or immunization. Even though HZ affects approximately one in three individuals in the United States, only one to two out of 10 patients with HZ develop HZO. Emerging data in children 0-10 years old reveal a significant decrease in the HZO incidence from 1994 to 2018, which may be explained by the incorporation of the two-dose VZV immunization schedule in the national vaccination programs worldwide [1].

Among HZO cases, 50%-71% develop ocular sequelae such as uveitis, stromal keratitis, and retinal necrosis. These complications have been attributed to either the direct effect of reactivated varicella virus, or a chronic active course of HZ infection or even a speculated immune-mediated mechanism. Approximately 2 out of 10 patients with HZO have a chronic disease course, necessitating long-term treatment [2]. Due to its rarity, evidence is scarce on the management of HZO in children, compared to adults, where there is an ongoing debate on the role of suppressive antiviral treatment to prevent recurrence [3]. Here we report a case of HZO caused by wild-type VZV strain in a previously healthy toddler fully immunized against VZV. Reviewing the literature on the management of pediatric HZO, we could not identify any formal guidelines regarding duration or secondary prophylaxis. In addition, there is a noticeable variability in treatment strategies applied as seen in the few published case reports.

## Case presentation

A previously healthy, three-year-old boy presented with fever and a five-day history of vesicular rash on the left forehead and left-eye conjunctivitis. The patient had been on topical corticosteroids and oral acyclovir for three days before admission, without improvement. Patient’s medical history was unremarkable and he was up to date on age-appropriate vaccinations, according to Greece’s national immunization program, where the second dose of the VZV vaccine is given at the age of three to five years. He had received the second dose of the VZV vaccine two months before presentation. No previous exanthematous illness was reported for the patient and his mother denied varicella-like illness during pregnancy.

**Figure 1:**
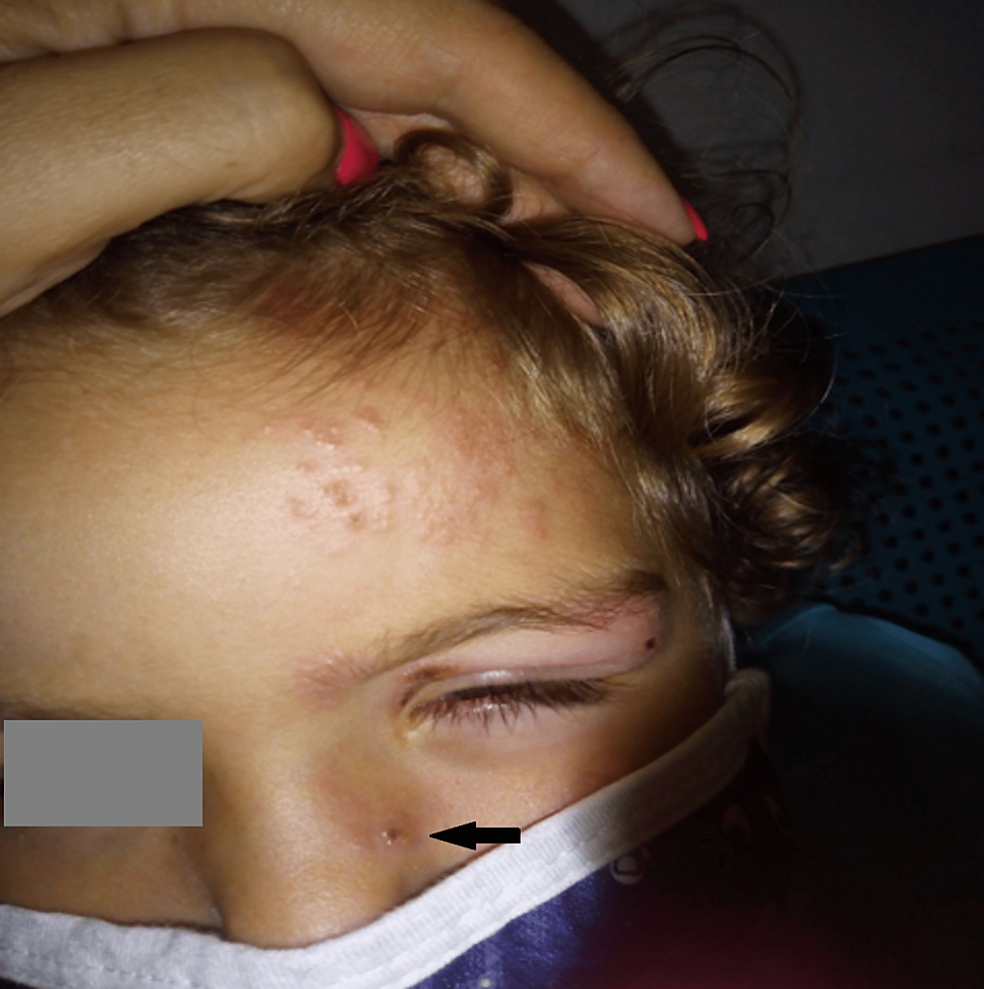
Clinical presentation of the patient, with the presence of Hutchinson's sign (black arrow)

On clinical examination, he had no central nervous system pathology. His Glasgow Coma Scale (GCS) score was 15/15; he had no meningeal rigidity and examination of his cranial nerves was intact. Hutchinson’s sign was present and mild anterior chamber inflammation was observed with conjunctival injection but no corneal epithelium defects (Figure 1).

Laboratory findings were unremarkable but the polymerase chain reaction (PCR) test on a crusted skin lesion revealed a wild-type varicella strain; serum VZV IgG titers were elevated. The patient showed no lymphopenia, and HIV and severe acute respiratory syndrome coronavirus 2 (SARS-CoV-2) serology tests were negative. Peripheral blood immunophenotyping was normal. Serum IgG, IgM and IgA levels were within the normal range, as were the immune response to tetanus and 13-valent pneumococcal vaccines (Table 1).

**Table 1:** Patient’s laboratory findings ALT: alanine transaminase, ANA: antinuclear antibodies, anti-dsDNA: anti-double-stranded DNA, AST: aspartate aminotransferase, Cl: chloride, CMV: cytomegalovirus, CRP: C-reactive protein, Hb: hemoglobin, HIV: human immunodeficiency virus, HSV: herpes simplex virus, Ht: hematocrit, Ig: immunoglobulin, K: potassium, LDH: lactate dehydrogenase, MCH: mean corpuscular hemoglobin, MCV: mean corpuscular volume, Na: sodium, PLT: platelets, RDW: red blood cell distribution width, VZV: varicella-zoster virus, WBC: white blood cells

Measurement	Reference range	Value
Complete blood count		
WBC (x10^3^/ml)	5.5-15.5	13
Neutrophils (x10^3^/ml)	1.5-8.5	2.73
Lymphocytes (x10^3^/ml)	2-8	8.97
Monocytes (x10^3^/ml)	0.3-0.7	1.04
Eosinophils (x10^3^/ml)	0.1-0.3	0.26
Hb (g/dl)	11.5-14.5	12.1
Ht (%)	33-43	36.3
MCV (fl)	73.6-87.5	79.8
MCH (pg)	25-31	26.7
PLT (x10^3^/ml)	150-300	448
Serum biochemical analysis		
CRP (mg/dl)	0-0.5	0.3
Glucose (mg/dl)	60-100	84
Urea (mg/dl)	5-20	32
Creatinine (mg/dl)	0.3-0.7	0.25
Na (mEq/l)	130-145	138
K (mEq/l)	4.1-5.3	4.5
Cl (mEq/l)	97-108	106
AST (IU/l)	15-46	31
ALT (IU/l)	8-36	14
LDH (IU/l)	150-300	214
Protein (g/dl)	5.6-8	6.8
Albumin (g/dl)	3-5.5	3.2
Serum immunoglobulins levels		
IgG (mg/dl)	441-1135	844
IgM (mg/dl)	47-200	104
IgA (mg/dl)	22-159	58.1
IgE (IU/ml)	0.19-16.9	255
ANA (IU/ml)	<1:40	Negative
Anti-dsDNA (IU/ml)	<200	Negative
Serology antibody tests		
HSV1 IgG (U)	<9	Negative
HSV1 IgM (U)	<9	Negative
HSV2 IgG (U)	<9	Negative
HSV2 IgM (U)	<9	Negative
VZV IgG (U)	<6	43
VZV IgM (U)	<9	Negative
CMV IgG (IU/l)	<6	Negative
CMV IgM (IU/l)	<0.85	Negative
HIV IgG (IU/l)	<0.9	Negative
HIV IgM (IU/l)	<0.9	Negative

Upon admission, treatment was switched from oral to intravenous administration of acyclovir (30 mg/kg/day), topical ganciclovir and azidamfenicol three times per day each for 10 days. Vesicles gradually crusted, but three days later, new-onset stromal opacities of the left cornea were noted and topical steroids were added three times per day (fluorometholone 0.1%). A complete ophthalmic examination revealed punctate keratitis with swelling of the anterior stroma, two foci of nummular keratitis, uveitis with marked anterior chamber reaction and conjunctivitis.

**Figure 2:**
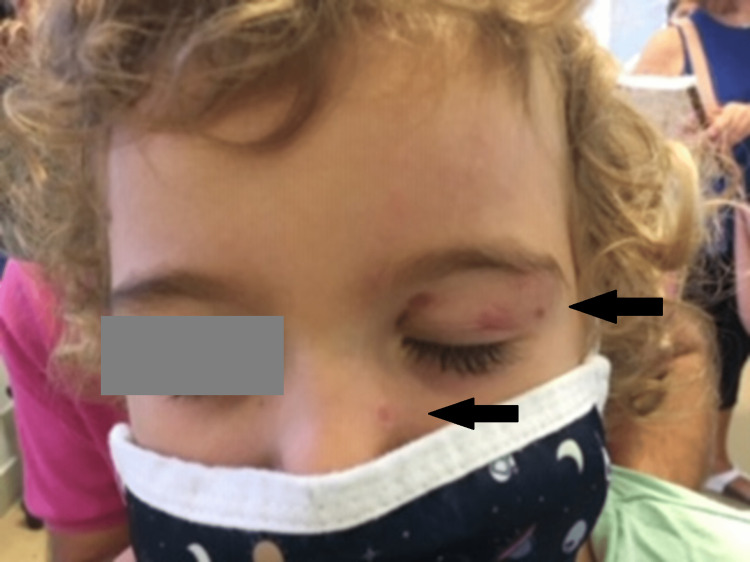
Clinical response 10 days after treatment initiation, with a significant improvement in the skin rash (black arrows)

The patient received 14 days of acyclovir with complete resolution of the skin rash and a remarkable improvement in the ocular findings (Figure 2). After that, he was switched to a prophylaxis dose (300 mg/m^2^/day), along with tapering of topical corticosteroids, for three months until all eye lesions had resolved. The patient has been on regular ophthalmological examination since then; one year later, his eye examination remains normal.

## Discussion

In countries where VZV immunization is widespread, HZ incidence in children is low (1/1000 person-years). That said, VZV latency may still be established following vaccination, due to either the vaccine strain itself (Oka strain) or the wild strain. In the latter case, zoster would be attributed to breakthrough varicella infection, or a subclinical course of infection prior to vaccination [2].

In a large survey enrolling 322 children with HZ, its incidence differed between vaccinated and unvaccinated subjects by 79% (48 vs. 230/100,000 person-years), especially in 3- to 9-year and 10- to 17-year age groups [4]. Interestingly, the HZ incidence was higher among vaccinated children aged 1-2 years old, compared to their non-vaccinated counterparts, a finding that could be explained by their incomplete VZV immunization status. In addition, 5% of subjects reported a recurrent HZ episode.

Patients with a high risk of HZO are those with primary or secondary immunodeficiency, primary varicella infection in the first year of life and/or intrauterine varicella exposure [2]. However, in a pan-German assessment of pediatric HZ hospitalizations, Grote et al. found that of the 244 children included in the study, 59% had no underlying immunodeficiency and they were more likely to suffer HZ complications than immunocompromised children (p<0.001) [5]. Notably, HZO was seen in 12% of children with HZ and was more commonly seen in immunocompetent compared to immunocompromised children (27% vs. 16%, p=0.39).

Due to the low incidence of HZO and the lack of randomized controlled trials (RCTs), some data on its management are extrapolated from herpes simplex virus disease, given the resembling latency and reactivation patterns of the two viruses. According to Kennedy et al., VZV establishes latency either by retrograde axonal transport or infected memory T-cells through a process called “round-trip infection”; VZV replicates in the neuron, travels anterograde to the skin and produces fresh lesions [6]. Therein, the virus travels back to the ganglia to infect more neurons. In HZO, although stromal keratitis or uveitis may not be due to active infection, a subclinical or intermittent viral shedding may possibly be the cause of subsequent inflammatory, anterior segment disease.

The Zoster Eye Disease Study (ZEDS) is an ongoing RCT for HZO in adults, comparing long-term valacyclovir suppressive treatment to placebo. In anticipation of its results, a survey among eye specialists showed that two-thirds of the participants agreed upon high-dose antivirals, especially valacyclovir and topical steroids for both recent-onset and chronic HZO [7]. In both cases, almost half of the investigators recommended the administration of antivirals for the duration of steroid treatment. Of note, 70% of the investigators believed that antiviral prophylaxis reduces chronicity and recurrences during the time of administration. However, 53% were uncertain whether secondary prophylaxis can act preventively after suppressive antiviral treatment discontinuation.

Currently, there is no consensus regarding the duration of antiviral treatment or the necessity for secondary prophylaxis in pediatric HZO. The German Dermatology Society recommends the maintenance of a sufficient virostatic plasma level with either high-dose acyclovir or valacyclovir, and highlights the need for a longer treatment duration in certain cases [8]. In addition, they suggest that systemic combination treatment with acyclovir and prednisolone should be balanced between the antiviral effect and the tissue-damaging immune reaction.

Miserochi et al. retrospectively studied 54 patients, 6-88 years old, with VZV ocular involvement, thus granting some insight into pediatric cases and management as well [9]. All participants received antivirals (acyclovir or valacyclovir) and/or steroids for a mean duration of 24.9±18.2 months. A total of 51% of patients suffered recurrences and 8.9% developed secondary corneal opacities regardless of aggressive corticosteroid co-administration.

Krall et al. reported a case of a six-year-old girl who presented with HZO that occurred one year after the varicella booster dose [10]. The girl received oral acyclovir, topical corticosteroids and cycloplegics for approximately one month. However, they were never able to wean off topical steroids due to HZO recurrence. Lin et al. reported a case of an adolescent with HZO and glaucoma eight years after VZV booster dose vaccination; the strain was not reported [11]. The patient received oral acyclovir, topical steroids and cycloplegics for approximately two months and was further treated for glaucoma, with a favorable outcome. Ivernizzi et al. presented a case of a six-year-old immunocompetent girl who presented with retinal vasculitis following acute granulomatous retinitis attributed to VZV reactivation, supported by an increase in her VZV IgG titer [12]. The patient received oral acyclovir, topical steroids and mydriatics for three months and was successfully treated.

Our patient was diagnosed with HZO during the COVID-19 pandemic. Nofal et al. presented four cases (two children, two adults) with HZO that developed over the course of SARS-CoV-2 infection [13]. All patients were immunocompetent and had a previous history of varicella infection. VZV reactivation was attributed to COVID-19-related lymphopenia and infection-induced stress. In our patient, the possibility of prior SARS-CoV-2 infection cannot be excluded given the often asymptomatic course in children. However, his SARS-CoV-2 antibody titers were absent on the 21st day post-HZO diagnosis.

## Conclusions

To conclude, we present a unique case of complicated HZO caused by a wild-type VZV strain in a previously healthy, VZV-vaccinated toddler. It is yet unknown whether antiviral prophylaxis is beneficial in children, how long it should last, and how it might affect the long-term prognosis. Whether a specific strain (Oka vs. wild type) or host immune response is responsible for the severe ocular sequelae in immunocompetent children needs to be further investigated. Studies that include children are needed in order to provide more evidence for proper HZO management.
